# Genetic polymorphisms in caveolin-1 associate with breast cancer risk in Chinese Han population

**DOI:** 10.18632/oncotarget.21560

**Published:** 2017-10-06

**Authors:** Meng Wang, Tian Tian, Xiaobin Ma, Wenge Zhu, Yan Guo, Zhao Duan, Jiangbo Fan, Shuai Lin, Kang Liu, Yi Zheng, Qianwen Sheng, Zhi-Jun Dai, Huixia Peng

**Affiliations:** ^1^ Department of Oncology, The Second Affiliated Hospital of Xi’an Jiaotong University, Xi’an, China; ^2^ Department of Biochemistry and Molecular Medicine, The George Washington University Medical School, Washington, DC, USA; ^3^ School of Life Science and Technology, Xi’an Jiaotong University, Xi’an, China; ^4^ Department of Obstetrics and Gynecology, The Second Affiliated Hospital of Xi’an Jiaotong University, Xi’an, China

**Keywords:** caveolin-1, polymorphism, breast cancer, risk

## Abstract

Caveolin-1(*CAV-1*) was demonstrated to be a tumor suppressor gene and be implicated in the development of breast cancer (BC). Numerous potentially functional polymorphisms in *CAV-1* have been identified, but their effects on BC were not clear. This case-control study aims to evaluate the relationship between *CAV-1* polymorphisms and BC risk. 560 BC patients and 583 healthy controls were enrolled in the present study, all from Chinese Han population. We detected 3 single nucleotide polymorphisms (rs3807987, rs1997623, and rs7804372) in *CAV-1* using the Sequenom MassARRAY method. The association between *CAV-1*genotypes and BC risk was assessed in six genetic models by calculating the odds ratio (OR) and 95% confidence intervals (95% CIs) with χ^2^-test. The *CAV-1* rs3807987 polymorphism was observed to increase the risk of BC And the A allele of rs3807987 relates to a larger tumor size (≥2cm) and lower incidence of PR-positive BC while the AA genotype of rs7804372 associates with a higher ER and Her-2 positive rate among BC patients. In addition, A_rs1997623_G_rs3807987_T_rs7804372_ haplotype was linked to a decreased risk of BC (OR =0.64, 95%CI=0.44-0.93), whereas C_rs1997623_A_rs3807987_T_rs7804372_ haplotype was related to an increased BC risk (OR =1.74, 95%CI=1.04-2.92). Our study suggests that *CAV-1* rs3807987 can increase the BC risk among Chinese Han women. And the rs3807987 and rs7804372 in *CAV-1* may serve as predictors for prognosis of BC.

## INTRODUCTION

Breast cancer (BC) is the most common cancer type and the main cause of cancer death in women all over the world [1]. In China, it also ranks the top of all the female cancers. In 2012, the morbidity and mortality of BC in China is about 0.221‰ and 0.054‰ respectively [2]. BC is an extremely heterogeneous disease. And multiple factors including hereditary and environmental factors are associated with its carcinogenesis. Genetic factors are believed to play a vital role in the development of BC. About 5-10% of all the BC cases are hereditary, caused by mutations in susceptibility genes [3].

*CAV-1* gene is located on human chromosome 7(7q31.1) and contains 3 exons. It encodes the protein caveolin-1, which is the essential structural component of caveolae [4, 5]. Caveolae is a special type of lipid raft, being rich in proteins and lipids. They are small vesicular invaginations of the plasma membrane in most cell types, especially in endothelial cells and adipocytes. And caveolae was shown to have several functions in signal transduction, cellular proliferation and differentiation, and contributes to tumorigenesis and tumor progression [6, 7]. Caveolin-1 has been implicated in the pathogenesis of cell transformation, oncogenesis and metastasis. Studies have shown that caveolin-1 can suppress breast cancer and its expression in BC tissue and cells were reduced compared with normal tissues [8, 9].

It has been indicated that *CAV-1* functions as a tumor suppressor gene and is mutated in up to 16-20% of breast cancers. It is also a inhibitor of the Ras-p42/44 mitogen-activated kinase pathway, which involves in cell cycle progression and was shown to be overexpressed in both primary and metastatic breast cancer cells [10, 11]. The loss of *CAV-1* can specifically up-regulate cyclin D1 and increase ERα expression, thus enhance estrogen-stimulated growth of BC cells [12]. All these evidence suggested that mutations in human *CAV-1* gene are involved in the BC onset and progression.

Single nucleotide polymorphism (SNP) is the most common form of mutation in the genome and is supposed to play a crucial role in genetic susceptibility to cancer. Numerous potentially functional SNPs in *CAV-1* have been identified through sequencing. Here, we chose 3 polymorphisms (rs3807987, rs1997623, and rs7804372) which are annotated in NCBI databases. These SNPs have been investigated in multiple cancer types including hepatocellular carcinoma, gastric cancer and prostate cancer. And they were found linked to cancer risk [13–15]. But their associations with BC susceptibility have not been fully understood. To investigate the relationship of *CAV-1* polymorphisms and BC risk, we conducted a case–control study in Chinese Han population.

## RESULTS

### Characteristics of study subjects

There were no significant differences in age, menopausal status, and procreative times between cases and controls(*P*>0.05). However, body mass index (BMI) values in two groups were significantly different. BMI values of BC patients were lower than those of healthy controls (*P*=0.038), which suggests that BMI may be a confounding factor. So, the results of additional analyses were adjusted for BMI. The detailed information of the subjects was described in our previous study [16].

### Association between *CAV-1* polymorphisms and BC risk

As shown in Table 1, the genotype distributions of the three *CAV-1* SNPs (rs3807987, rs1997623, and rs7804372) in the control group were all conformed to HWE (*P*=0.111, 0.356 and 0.125 respectively). The rs3807987 polymorphism was related to increased BC risk in the heterozygous, dominant, and overdominant models (GA *vs.* GG: OR=1.41, 95%CI=1.09-1.82, *P*=0.008; GA+AA *vs.* GG: OR= 1.36, 95%CI= 1.07-1.74, *P*=0.013; GA *vs.* GG + AA: OR=1.40, 95%CI=1.09-1.81, *P*=0.008). Whereas no obvious relationship was found between the other two polymorphisms and BC risk. We further conducted subgroup analyses by age and menopausal status. However, neither of the polymorphisms was found to associate with BC risk in any genetic models (Supplementary Table 1 and Supplementary Table 2).

**Table 1:** Genotype frequencies of *Cav-1* polymorphism in cases and controls

Model	Genotype	Cases (n,%)	Control (n,%)	*P*^†^	OR (95% CI)	*P* (HWE)
**rs3807987 (G14713A)**						
**Co-dominant**	GG	345 (61.61%)	400 (68.61%)		1.00	0.111
Heterozygote	GA	193 (34.46%)	159 (27.27%)	**0.008**	**1.41(1.09-1.82)**	
Homozygote	AA	22 (3.93%)	24 (4.12%)	0.841	1.06 (0.59-1.93)	
**Dominant**	GG	345 (61.61%)	400 (68.61%)		1.00	
	GA+AA	215 (38.39%)	183 (31.39%)	**0.013**	**1.36 (1.07-1.74)**	
**Recessive**	GG+GA	538 (96.07%)	559 (95.88%)		1.00	
	AA	22 (3.93%)	24 (4.12%)	0.872	0.95 (0.53-1.72)	
**Overdominant**	GG + AA	367 (65.54%)	424 (72.73%)		1.00	
	GA	193 (34.46%)	159 (27.27%)	**0.008**	**1.40 (1.09-1.81)**	
**Allele**	G	883 (78.84%)	959 (82.25%)		1.00	
	A	237 (21.16%)	207 (17.75%)	0.040	1.24 (1.01-1.53)	
**rs1997623 (C239A)**						
**Co-dominant**	CC	507 (90.54%)	509 (87.31%)		1.00	0.356
Heterozygote	CT	51 (9.11%)	70 (12.01%)	0.107	0.73 (0.50-1.07)	
Homozygote	TT	2 (0.36%)	4 (0.67%)	0.418	0.50 (0.09-2.75)	
**Dominant**	CC	507 (90.54%)	509 (87.31%)		1.00	
	CT+TT	53 (9.46%)	74 (12.69%)	0.083	0.72 (0.50-1.04)	
**Recessive**	CC+CT	558 (99.64%)	579 (99.31%)		1.00	
	TT	2 (0.36%)	4 (0.67%)	0.442	0.52 (0.10-2.84)	
**Overdominant**	CC+TT	509 (90.89%)	513 (91.61%)		1.00	
	CT	51 (9.11%)	70 (12.01%)	0.111	0.73 (0.50-1.08)	
**Allele**	C	1065 (95.09%)	1088 (93.31%)		1.00	
	T	55 (4.91%)	78 (6.69%)	0.069	0.72 (0.51-1.02)	
rs7804372^*^ **(T29107A)**						
**Co-dominant**	TT	317 (56.61%)	338 (58.08%)		1.00	0.125
Heterozygote	AT	207 (36.96%)	202 (34.71%)	0.482	1.09(0.85-1.40)	
Homozygote	AA	36 (6.43%)	42 (7.22%)	0.708	0.91 (0.57-1.46)	
**Dominant**	TT	317 (56.61%)	338 (58.08%)		1.00	
	AT+AA	243 (43.39%)	244 (41.92%)	0.616	1.06 (0.84-1.34)	
**Recessive**	TT+AT	524 (93.57%)	540(92.78%)		1.00	
	AA	36 (6.43%)	42 (7.22%)	0.598	0.88 (0.56-1.40)	
**Overdominant**	TT+AA	353 (63.04%)	380 (65.29%)		1.00	
	AT	207 (36.96%)	202 (34.71%)	0.427	1.10 (0.87-1.41)	
**Allele**	T	841 (75.09%)	878 (75.43%)		1.00	
	A	279 (24.91%)	286 (24.57%)	0.851	1.018 (0.84-1.23)	

^†^ Adjusted for age and body mass index. rs7804372^*^: controls missing, n = 1.

### *CAV-1* polymorphisms and clinicopathological features of BC

We also investigated the relationship of *CAV-1* SNPs with clinical and histological features of BC, including tumor size, lymph node metastasis, and the statuses of estrogen receptor (ER), progestogen receptor (PR), human epidermal growth factor receptor 2 (Her-2), and Ki67. As for rs3807987, we found that the A allele was related to a larger tumor size (≥2cm) in BC patients (GA+AA *vs.* GG: OR= 1.52, 95%CI= 1.05-2.21; A *vs.* G: OR= 1.48, 95%CI=1.07-2.03), and lower incidence of PR-positive breast cancer (GA+AA *vs.* GG: OR= 0.69, 95%CI= 0.49-0.98; A *vs.* G: OR= 0.72, 95%CI= 0.54- 0.96) (Table 2). For rs7804372, BC patients with AA genotype are more likely to have ER- positive tumors than TT and TA genotype carriers (recessive model: OR =2.15, 95%CI=1.02-4.54). Moreover, the AA genotype of rs7804372 associates with a higher Her-2 positive rate compared with TT and TT+TA genotypes (Table 3). No significant association exists between the rs1997623 polymorphism and any of the clinical parameters (Supplementary Table 3).

**Table 2:** The associations between the *Cav-1* rs3807987 polymorphism and clinical characteristics of breast cancer

Variables	GG	GA	AA	Heterozygote Adjusted OR (95% CI)	Homozygote Adjusted OR (95% CI)	Dominant Adjusted OR (95% CI)	Recessive Adjusted OR (95% CI)	Allele Adjusted OR (95% CI)
**Tumor size**								
<2 cm	128	56	4	1.00				
≥2 cm	217	137	18	1.44(0.99-2.11)	2.65(0.88-8.02)	**1.52(1.05-2.21)**	2.34(0.78-7.01)	**1.48(1.07-2.03)**
**LN metastasis**								
Negative	151	74	11	1.00				
Positive	194	119	11	1.25(0.87-1.79)	0.78(0.33-1.84)	1.19(0.84-1.68)	0.72(0.31-1.69)	1.09(0.81-1.46)
**ER**								
Negative	147	92	8	1.00				
Positive	198	101	14	0.82(0.5721.16)	1.30(0.53-3.18)	0.85(0.61-1.20)	1.40(0.58-3.39)	1.13(0.85-1.52)
**PR**								
Negative	145	97	13	1.00				
Positive	200	96	9	0.72(0.50-1.02)	0.50(0.21-1.21)	**0.69(0.49-0.98)**	0.57(0.24-1.35)	**0.72(0.54-0.96)**
**Her-2**								
Negative	242	132	15	1.00				
Positive	103	61	7	1.09(0.74-1.59)	1.10(0.43-2.77)	1.09(0.75-1.57)	1.06(0.43-2.67)	1.07(0.78-1.46)
**Ki67**								
< 14%	116	69	10	1.00				
≥14%	229	124	12	0.91(0.63-1.32)	0.61(0.26-1.45)	0.87(0.61-1.24)	0.63(0.27-1.48)	0.86(0.64-1.16)

OR: odds ratio; CI: confidence interval; LN: lymph node; ER: estrogen receptor; PR: progesterone receptor; Her-2: human epidermal growth factor receptor-2.

**Table 3:** The associations between the *Cav-1* rs7804372 polymorphism and clinical characteristics of breast cancer

Variables	TT	AT	AA	Heterozygote Adjusted OR (95% CI)	Homozygote Adjusted OR (95% CI)	Dominant Adjusted OR (95% CI)	Recessive Adjusted OR (95% CI)	Allele Adjusted OR (95% CI)
**Tumor size**								
<2 cm	103	70	15	1.00				
≥2 cm	214	137	21	0.942(0.650-1.366)	0.674(0.334-1.361)	0.895(0.628-1.274)	0.690(0.347-1.372)	0.874(0.658-1.161)
**LN metastasis**								
Negative	140	77	19	1.00				
Positive	177	130	17	1.335(0.933-1.911)	0.708(0.355-1.412)	1.211(0.862-1.701)	0.632(0.321-1.245)	1.052(0.799-1.385)
**ER**								
Negative	141	96	10	1.00				
Positive	176	111	26	0.926(0.652-1.317)	2.083(0.972-4.464)	1.035(0.739-1.450)	**2.147(1.015-4.542)**	1.147(0.872-1.509)
**PR**								
Negative	134	101	20	1.00				
Positive	183	106	16	0.768(0.540-1.093)	0.586(0.293-1173)	0.738(0.528-1.033)	0.651(0.330-1.283)	0.765(0.583-1.004)
**Her-2**								
Negative	216	155	18	1.00				
Positive	101	52	18	1.717(0.484-1.063)	**2.139(1.068-4.284)**	0.865(0.601-1.246)	**2.425(1.229-4.786)**	1.065(0.795-1.426)
**Ki67**								
< 14%	114	72	9	1.00				
≥14%	203	135	27	1.053(0.730-1.519)	1.685(0.766-3.707)	1.123(0.790-1.597)	1.651(0.760-3.585)	1.205(0.873-1.553)

OR: odds ratio; CI: confidence interval; LN: lymph node; ER: estrogen receptor; PR: progesterone receptor; Her-2: human epidermal growth factor receptor-2.

### Association between *CAV-1* haplotypes and BC risk

To evaluate the effect of SNPs interaction on BC risk, we performed haplotype analysis. The allele distributions and frequencies of the *CAV-1* haplotypes are presented in Table 4, and the most frequent haplotype in controls was chosen as a reference. We observed that the A_rs1997623_G_rs3807987_T_rs7804372_ haplotype was associates with a reduced BC risk (OR =0.64, 95%CI=0.44-0.93, *P*=0.018) compared with the C_rs1997623_G_rs3807987_T_rs7804372_ wild type, whereas the C_rs1997623_A_rs3807987_T_rs7804372_ haplotype was related to a higher risk of BC (OR =1.74, 95%CI=1.04-2.92, *P*= 0.033). No relationship was found between other haplotypes and BC risk.

**Table 4:** The haplotype analysis of *Cav-1* polymorphisms and breast cancer risk

Haplotypes			Cases (N=1120) n, %	Controls (N=1166) n, %	OR (95% CI)	*p*
rs1997623	rs3807987	rs7804372				
C	G	T	746 (66.61%)	779(66.81%)	1.00 (reference)	
C	A	A	189(16.88%)	183(15.69%)	1.08(0.86-1.35)	0.514
C	G	A	90(8.04%)	102(8.75%)	0.92 (0.68-1.25)	0.594
A	G	T	47 (4.20%)	77(6.60%)	**0.64(0.44-0.93)**	**0.018**
C	A	T	40(3.57%)	24(2.06%)	**1.74(1.04-2.92)**	**0.033**
A	A	T	8(0.71%)	-	-	-
A	G	T	-	1(0.09%)	-	-

## DISCUSSION

Caveolin-1, encoded by *CAV-1* gene, is a 21- to 24-kDa integral membrane protein. It is enriched in specialized plasma membranes invaginations called caveolae, which are found in many cell types. As an intracellular structural protein, caveolin-1 regulates several important signaling transduction related to BC including ER, EGFR, Her2/neu, TGF-β, and mTOR pathways [5, 17]. Since genetic factors play a crucial role in human breast carcinogenesis, polymorphisms in genes that encode proteins involved in the BC development can act as biomarkers and may even turn out to be useful targets for particular therapeutic approaches to BC [3]. Hence, we designed this study to investigate the association of *CAV-1* polymorphisms with BC susceptibility.

A number of studies have explored the associations between *CAV-1* genetic polymorphisms and the risk of various cancer types including hepatocellular carcinoma [13], gastric cancer [14], esophageal cancer [18], colorectal cancer [19], renal cell carcinoma [20], prostate cancer [15], bladder cancer [21] and leukemia [22]. We also found one study investigated the effect of CAV-1 SNPs on BC susceptibility by Liu et al. In Liu’s study, they determined the six SNPs in *CAV-1* (rs1997623, rs3807987, 12672038, rs3757733, rs7804372, and rs3807992) and assessed their association with BC risk. The results indicated that the genotype distributions of rs3807987 and rs7804372 were significantly different in BC patients and healthy controls. The GG/AT or GG/AA haplotypes associated with a lower risk of BC (OR=0.69, 95% CI=0.57-0.92), whereas the AG/TT haplotype conferred a higher risk of BC (OR=1.50, 95% CI=1.14-2.12)[23]. The present study has some difference with Liu’s study. First, we assessed three SNPs in *CAV-1* which Liu et al. also investigated, but the results were different. Our study showed that only rs3807987 related to BC susceptibility. The GA or GA+AA genotypes of rs3807987 may increase the risk of BC compared with GG or GG+AA genotypes. The difference may be due to sample size and divergence of the population. Liu et al. investigated a Taiwanese population, whereas the volunteers in our study subjects all come from northwest of China. Second, we further explored the association of *CAV-1* polymorphisms and clinical features of BC. Our results suggested that rs3807987 polymorphism in *CAV-1* was associated with tumor size and PR status while rs7804372 polymorphism was linked to ER and Her-2 status. The underlying mechanisms for this finding are unknown by now, which can be a promising field for future studies. We also performed stratified analyses by age and menopausal status, though there was no significant difference in the subgroups.

Previous studies have demonstrated that the expression of *CAV-1* in both mRNA and protein levels is down-regulated in breast cancer and *CAV-1* re-expression can inhibit the growth and the invasive and migratory potential of BC cells [12, 24]. In addition, the decreased expression of caveolin-1 in BC is significantly associated with advanced tumor stage, invasion or metastasis, early recurrence, and poor outcome [5]. The underlying mechanism is not fully elucidated. Some studies suggested that mutational *CAV-1* can induce cellular transformation, activate MAPK signaling pathway and alter actin networks in BC cells, thus promote invasion-ability of BC. And aberrant promoter methylation of *CAV-1* may also play a role in the regulation of BC onset [9, 25]. Our study proved the role of *CAV-1* in breast cancer susceptibility and revealed its relation with clinical characteristic of BC. However, there are some limitations in our study should be noticed. Firstly, selection bias may exist since this is a hospital-based case-control study and all the subjects were from the same hospital. Secondly, the sample size was inadequate for a stratified analysis of BC subtypes. Thirdly, the effect of other predisposing factors such as family heredity history, environmental exposures, alcohol consumption, and lifestyle was not assessed due to lack of data. Besides, our study is a clinical research and lacks functional experiments to support the results. Thus, future population-based studies are needed to assess these factors as well for a more accurate evaluation of the effect of *CAV-1* genetic polymorphisms on BC risk. And, the functions of these polymorphisms and the underlying mechanisms need to be explored in subsequent studies.

In conclusion, the current study showed that *CAV-1* polymorphism rs3807987 associates with BC susceptibility in Chinese Han population. The C_rs1997623_A_rs3807987_T_rs7804372_ haplotype of *CAV-1* may increase the risk of BC whereas the A_rs1997623_G_rs3807987_T_rs7804372_ haplotype may be protective for the onset of BC. Moreover, *CAV-1* rs3807987 and rs7804372 polymorphisms were related to tumors size and ER/PR /Her-2 status, which may act as predictors for BC prognosis and effect of treatment. Further large prospective studies and functional studies are needed in order to provide more evidence about the influence of *CAV-1* genetic variants on BC risk, and to explore the possible molecular mechanism.

## MATERIALS AND METHODS

### Study subjects

This study was approved by the Ethics Committee of the Second Affiliated Hospital of Xi’an Jiaotong University (Xi’an, China). Patients with BC were selected from the Department of Oncology. At the mean time, healthy individuals who came to the same hospital for a checkup were recruited as controls. All the cases were confirmed by histology or pathology and the controls were matched based on age and menopausal status. Patients who had received chemotherapy or radiotherapy before surgery or had another type of cancer were excluded [26, 27]. Finally, 1143 subjects (560 cases and 583 controls) were enrolled in the study. All the subjects were unrelated Chinese Han females. They were well informed of the purpose of our study and all of them signed a consent form.

### DNA extraction and genotyping

The blood samples of all the subjects were collected in EDTA- coating tubes and stored at−80°C for further use. Genomic DNA was extracted from peripheral blood samples with the Universal Genomic DNA Extraction Kit (version 3.0; TaKaRa Bio Inc., Kusatsu, Japan) following the manufacturer’s instructions. DNA quantity was evaluated by spectrometry (DU530 UV/VIS spectrophotometer, Beckman Instruments, Fullerton, CA). Tree tag-SNPs (rs3807987, rs1997623, and rs7804372) were selected in our study using the data from HapMap database. We only considered SNPs of which minor allele frequency (MAF) was greater than 0.01. A multiplexed SNP MassEXTEND assay was designed by the Sequenom MassARRAY Assay Design 3.0 Software (Agena Bioscience, Inc., San Diego, CA). SNP genotyping was performed by the SequenomMassARRAY RS1000. Primers for the three SNPs are shown in Table 5. Data was analysed by Sequenom Type 4.0 Software (Sequenom, Inc).

**Table 5:** Primers used for this study

SNP_ID	1st-PCRP	2nd-PCRP	UEP_SEQ
rs3807987	ACGTTGGATGTGTGGC ATCCAGATGATCTC	ACGTTGGATGGAATT TGCCTGTCCTGTCTG	CTGTCTGCCAGACAC
rs1997623	ACGTTGGATGGTGAA G AGAAGCCAGGAATG	ACGTTGGATGCTTT TCCGTGTCCTCCTAGC	CCCTTAGCTCAGGGGC TCCC
rs7804372	ACGTTGGATGCTGAA TCCAACAAAGCTCGG	ACGTTGGATGAAT CTGCTGAAGCAGCTGTG	GTGTGCTTTGATTGATG TGGA

PCRP: polymerase chain reaction primer; SNP:single nucleotide polymorphism; UEP-SEQ: unextension primer sequence.

### Statistical analysis

The allele and genotype frequencies of *CAV-1* polymorphisms were counted directly. And the Hardy-Weinberg equilibrium (HWE) for each SNP in the control group was examined using Pearson χ^2^-test. The differences of allele and genotype distribution for each SNP between cases and controls were also determined by Pearson χ^2^-test while the differences in clinical characteristics were assessed by Student t-test or χ^2^-test. A two-sided P-value < 0.05 was considered statistically significant in all the tests. Associations of *CAV-1* polymorphisms with BC risk and the patients’ clinical characteristics were estimated with an odds ratio (OR) and 95% confidence interval (CI) in the codominant model (homozygous model: aa *vs.* AA; heterozygous model: Aa *vs.* AA), the dominant model (AA *vs.* Aa+aa), the recessive model (aa *vs.* AA+Aa), the overdominant model (Aa *vs.* AA+ aa), and the allele model (a *vs.* A) respectively (A: the major allele, a: the minor allele). And logistic regression analysis was used to adjust for confounding factors. PHASE v2.1 software was used to perform the haplotype analysis. All the statistical analyses were accomplished using SPSS 22.0 software (IBM Corporation, NY, USA).

## SUPPLEMENTARY MATERIALS TABLES



## Abbreviations

CAV-1: caveolin-1
BC: breast cancer
SNP: single nucleotide polymorphism
OR: odds ratio
95% CI: 95% confidence interval
BMI: body mass index
HWE: Hardy-Weinberg equilibrium
LN: lymph node
ER: estrogen receptor
PR: progesterone receptor
Her-2: human epidermal growth factor receptor-2
