# Supplementary material for: Genetic polymorphisms in caveolin-1 associate with breast cancer risk in Chinese Han population
Source: Oncotarget. 2017 Oct 6;8(53):91654–61. doi: 10.18632/oncotarget.21560 (PMC5710954; doi:10.18632/oncotarget.21560)
Supplement: Supplementary file 1 [file oncotarget-08-91654-s001.pdf]

Genetic polymorphisms in caveolin-1 associate with breast cancer risk in Chinese Han population

SUPPLEMENTARY MATERIALS

Supplementary Table 1: Association between Cav-1 SNPs and age of breast cancer patients

Model	Genotype	Age<49			≥49		
		Cases	Control	Adjusted OR (95% CI)	Cases	Control	Adjusted OR (95% CI)
**G14713A rs3807987**							
**Co-dominant**	GG	180	213	1.00	165	187	1.00
Heterozygote	GA	101	85	1.406(0.991-1.996)	92	74	1.409(0.972-2.041)
Homozygote	AA	13	13	1.183(0.535-2.618)	9	11	0.927(0.375-2.293)
**Dominant**	GG	180	213	1.00	165	187	1.00
	GA+AA	114	98	1.377(0.985-1.924)	101	85	1.347(0.943-1.923)
**Recessive**	GG+GA	281	298	1.00	257	261	1.00
	AA	13	13	1.606(0.483-2.327)	9	11	0.831(0.339-2.039)
**Allele**	G	461	511	1.00	422	448	1.00
	A	127	111	1.268(0.955-1.685)	110	96	1.216(0.897-1.649)
**C239A rs1997623**							
**Co-dominant**	CC	258	261	1.00	249	248	1.00
Heterozygote	CT	34	47	0.732(0.456-1.175)	17	23	0.736(0.384-1.412)
Homozygote	TT	2	3	0.674(0.112-4.070)	0	1	1.004(0.996-1.012)
**Dominant**	CC	258	261	1.00	249	248	1.00
	CT+TT	36	50	0.728(0.459-1.156)	17	24	0.705(0.370-1.346)
**Recessive**	CC+CT	292	308	1.00	266	271	1.00
	TT	2	3	0.703(0.117-4.239)	0	1	1.004(0.996-1.011)
**Allele**	C	550	569	1.00	515	519	1.00
	T	38	54	0.728(0.473-1.121)	17	25	0.685(0.366-1.284)
**T29107A rs7804372***							
**Co-dominant**	TT	158	178	1.00	159	160	1.00
Heterozygote	AT	109	99	1.240(0.877-1.754)	98	103	0.957(0.673-1.363)
Homozygote	AA	27	33	0.922(0.531-1.601)	9	9	1.006(0.389-2.601)
**Dominant**	TT	158	178	1.00	159	160	1.00
	AT+AA	136	132	1.161(0.842-1.601)	107	112	0.961(0.682-1.356)
**Recessive**	TT+AT	267	277	1.00	257	263	1.00
	AA	27	33	0.849(0.497-1.450)	9	9	1.023(0.400-2.619)
**Allele**	T	425	455	1.00	416	423	1.00
	A	163	165	1.058(0.821-1.363)	116	121	0.975(0.731-1.301)

rs7804372*: controls missing, n = 1.

Supplementary Table 2: Association between Cav-1 SNPs and menopausal status of breast cancer patients

Model	Genotype	Premenopausal			Postmenopausal		
		Cases	Control	Adjusted OR (95% CI)	Cases	Control	Adjusted OR (95% CI)
**G14713A rs3807987**							
**Co-dominant**	GG	171	202	1.00	174	198	1.00
Heterozygote	GA	85	69	1.455(0.998-2.122)	108	90	1.366(0.966-1.930)
Homozygote	AA	8	10	0.945(0.365-2.448)	14	14	1.138(0.528-2.453)
**Dominant**	GG	171	202	1.00	174	198	1.00
	GA+AA	93	79	1.391(0.968-1.998)	122	104	1.335(0.958-1.860)
**Recessive**	GG+GA	256	271	1.00	282	288	1.00
	AA	8	10	0.847(0.329-2.179)	14	14	1.021(0.478-2.181)
**Allele**	G	427	473	1.00	456	486	1.00
	A	101	89	1.257(0.919-1.720)	136	118	1.228(0.930-1.622)
**C239A rs1997623**							
**Co-dominant**	CC	233	239	1.00	274	270	1.00
Heterozygote	CT	30	39	0.789(0.474-1.313)	21	31	0.668(0.374-1.191)
Homozygote	TT	1	3	0.342(0.035-3.311)	1	1	0.985(0.061-15.835)
**Dominant**	CC	233	239	1.00	274	270	1.00
	CT+TT	31	42	0.757(0.460-1.246)	22	32	0.677(0.384-1.196)
**Recessive**	CC+CT	263	278	1.00	296	301	1.00
	TT	1	3	0.352(0.036-3.409)	1	1	1.020(0.064-16.389)
**Allele**	C	496	517	1.00	569	571	1.00
	T	32	45	0.741(0.463-1.186)	23	33	0.699(0.406-1.206)
**T29107A rs7804372***							
**Co-dominant**	TT	149	155	1.00	168	183	1.00
Heterozygote	AT	100	102	1.020(0.714-1.456)	107	100	1.166(0.826-1.644)
Homozygote	AA	15	23	0.678(0.341-1.350)	21	19	1.204(0.625-2.318)
**Dominant**	TT	149	155	1.00	168	183	1.00
	AT+AA	115	125	0.957(0.682-1.343)	128	119	1.172(0.846-1.623)
**Recessive**	TT+AT	249	257	1.00	275	283	1.00
	AA	15	23	0.673(0.343-1.320)	21	19	1.137(0.598-2.162)
**Allele**	T	398	412	1.00	443	466	1.00
	A	130	148	0.909(0.692-1.195)	149	138	1.136(0.871-1.481)

rs7804372*: controls missing, n = 1.

Supplementary Table 3: The associations between the *Cav-1* rs1997623 Polymorphism and clinical characteristics of breast cancer

Variables	CC	CA	AA	Heterozygote Adjusted OR (95% CI)	Homozygote Adjusted OR (95% CI)	Dominant Adjusted OR (95% CI)	Recessive Adjusted OR (95% CI)	Allele Adjusted OR (95% CI)
**Tumor size**								
<2 cm	170	17	1			1.00		
≥2 cm	337	34	1	1.01(0.55-1.86)	0.50(0.03-8.11)	0.98(0.54-1.78)	0.69(0.04-11.10)	0.96(0.54-1.69)
**LN metastasis**								
Negative	215	21	0			1.00		
Positive	292	30	2	1.05 (0.59-1.89)	-	1.12(0.63-2.00)	-	1.19(0.68-2.08)
**ER**								
Negative	221	25	1			1.00		
Positive	286	26	1	0.80(0.45-1.43)	0.77(0.05-12.42)	0.80(0.46-1.41)	0.79(0.05-12.67)	0.81(0.47-1.39)
**PR**								
Negative	226	28	1			1.00		
Positive	281	23	1	0.66(0.37-1.18)	0.80(0.05-12.93)	0.67(0.38-1.18)	0.84(0.05-13.42)	0.68(0.40-1.18)
**Her-2**								
Negative	356	31	2			1.00		
Positive	151	20	0	1.52(0.84-2.75)	-	1.43(0.79-2.57)	-	1.32(0.75-2.32)
**Ki67**								
< 14%	179	15	1			1.00		
≥14%	328	36	1	1.310(0.698-2.457)	0.55(0.03-8.78)	1.26(0.68-2.33)	0.53(0.03-8.57)	1.21(0.67-2.16)

OR: odds ratio; CI: confidence interval; LN: lymph node; ER: estrogen receptor; PR: progesterone receptor; Her-2: human epidermal growth factor receptor-2.
